# Incomplete Kawasaki disease in Egypt

**DOI:** 10.21542/gcsp.2017.24

**Published:** 2017-10-31

**Authors:** Hala M. Agha, Hala S. Hamza

**Affiliations:** Department of Pediatrics, Pediatric Cardiology Division, Specialized Pediatric Hospital, Cairo University

## Introduction

Kawasaki disease (KD) is a hybrid condition at the junction of infectious diseases, immunology, rheumatology, and cardiology.^1^ KD is a systemic vasculitis of unknown etiology predominately affecting medium-sized vessels such as the coronary arteries, which mainly affects infants and children^2^. The disease itself may be the characteristic manifestation of a common pathway of immune-mediated vascular inflammation in genetically susceptible hosts^3^. Untreated KD may lead to the formation of coronary artery aneurysms and sudden cardiac death in children. The diagnosis of KD is based on the clinical features of fever of at least 5 days together with at least 4 or 5 other features including rash, bilateral conjunctival injection, changes in peripheral extremities, lymphadenopathy and oropharyngeal changes^4^. The diseases that must be differentiated from KD because of similar clinical findings include viral infections (measles, adenovirus, enterovirus, and Epstein-Barr virus), scarlet fever, staphylococcal scaled skin syndrome, toxic shock syndrome, polyarteritis nodosa, bacterial cervical lymphadenitis, and juvenile rheumatoid arthritis^5,6^. Because each of the symptoms commonly occurs in other childhood illnesses, the disease can be difficult to diagnose, especially in children who present with an incomplete form of the disease. KD has not been previously reported from Egypt and there are special challenges in recognizing complete KD in a country where physicians have limited experience with the disease. The diagnosis of incomplete KD is thus even more challenging in this setting.

## What are the diagnostic criteria of incomplete KD?

The term ‘incomplete’ has been used to describe patients with incomplete presentation, regardless of the presence of coronary complications^8,9^. According to the diagnostic criteria of incomplete KD established by the AHA, children >6 months of age with incomplete presentation might have unexplained fever for >5 days associated with 2 or 3 of the principles features^5^. The AHA recommended a diagnostic algorithm of incomplete KD which comprises 6 supplemental laboratory and echocardiographic criteria. More than 3 laboratory criteria support the diagnosis of atypical KD (Table 1)^5^.

**Table 1:** Supplemental laboratory and echocardiographic criteria for the diagnosis of incomplete Kawasaki disease prepared by the American Heart Association^5^.

**A: Laboratory Criteria**
Serum albumin ≤3g/dl
Anemia for age
Elevation of alanine aminotransferase
Platelets after 7days ≥450,000/mm^3^
WBC ≥15,000/mm^3^
Urine WBC ≥10/HPF
**B: Echocardiographic Criteria**
Z score of LAD or RCA ≥2.5
Coronary arteries meet Japanese Ministry of Health criteria for aneurysm:
Internal lumen diameter
>3 mm in children <5 years old, or
>4 mm in children >5 years old
Of a segment measures ≥1.5times that of an adjacent segment
Clearly irregular coronary lumen
Other 6 suggestive features (if >3features, positive)
Perivascular brightness of coronary arteries
Lack of tapering of coronary arteries
Decreased LV function
Mitral regurgitation
Pericardial effusion
Z score in LAD or RCA of 2 to 2.5

## Case Report 1

**Figure 1.**
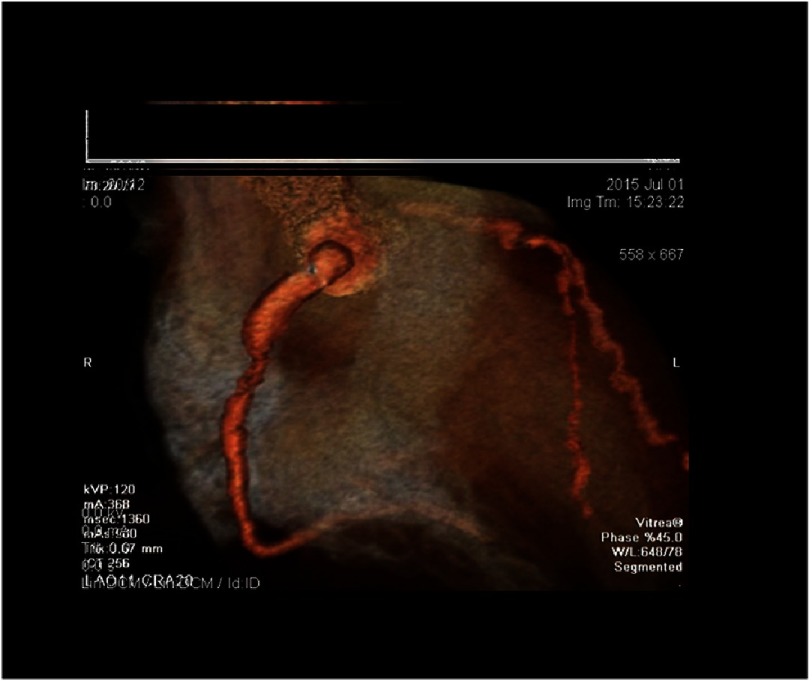
The proximal RCA of case 1 shows a large aneurysm, measuring about 23 mm long and 11 × 8.7 mm in diameter. There is stenosis at the mid-segment of the RCA. The rest of the RCA appears of normal caliber.

A 4-year-old female presented with prolonged fever for 1month which was misdiagnosed as chicken-pox. She had non purulent conjunctivitis and peri-ungual desquamation. Echocardiography revealed coronary artery aneurysms; RCA and LCA measuring around 9 mm and 6 mm respectively. There was improvement of her clinical status and disappearance of fever after she received the second dosage of intravenous immunoglobulin (IVIG) on 2g/kg. During her follow-up by echocardiography for 4 years, there was mild regression of the coronary aneurysms compared to that of the initial measurements. Multi-slice CT angiogram showed regression of the LAD and RCA aneurysms with appearance of LAD and RCA stenosis (Figure 1).

Coronary angiography, intravascular ultrasound (IVUS) and fractional flow reserve (FFR) were decided and revealed proximal RCA aneurysm partially occluded by organized thrombi with the inlet and outlet of the aneurysm showing 50% and 90% stenoses respectively, with significantly reduced FFR (0.78 resting gradient without giving adenosine) across both lesions. Proximal LAD smaller aneurysm and mid LAD lesion with a 50% stenosis, these findings are due to thrombi with adequate FFR (0.88 after intra-coronary adenosine).

*This case underscores the following issues in the diagnosis of acute KD:*

- A long time between the onset of symptoms and diagnosis (1 month)
- Persistence of 2 findings; non purulent conjunctivitis and peri-ungual desquamation
- Delay in diagnosis led to large coronary aneurysms in both coronary arteries.

## Case Report 2

A 3-year-old boy was referred to our clinic because of high-grade fever and jaundice. The onset of fever was from 7 days followed by icteric tinge and dark urine manifested 2 days after the onset of fever and resolved after 3 days.

History obtained from the parents revealed no hepatotoxic drug consumption or maculopapular rash. On physical examination, body temperature was 39°C and desquamation on the tips of fingers and toes were seen. Eye examination showed bilateral bulbar conjunctival injection. Abdominal examination revealed no hepatosplenomegaly or tenderness. Abdominal ultrasonography revealed no hepatosplenomegaly with normal echogenecity, gallbladder and bile ducts were normal and ascites was not detected.

Laboratory findings showed elevation of ESR, CRP and liver transaminases with hyperbilirubinemia. There was no serologic finding compatible with viral hepatitis (Table 2).

**Table 2:** Laboratory characteristics of the patient.

White blood cells	10,000/mm^3^
Hemoglobin level	10g/dl
Platelets	480,000/ mm^3^
ESR	100
CRP	24 mg/dl
AST	60 IU/L
ALT	70 IU/L
Bilirubin (Total, Direct)	(4 mg/dl, 1.5 mg/dl)
Serum Albumin	3 g/dl
Prothrombin time	13 sec
HBSAg	Negative
Anti HAV(IgM)	Negative
Anti HCV	Negative

According to clinical and laboratory findings of this patient, KD was suspected and echocardiography findings were normal. Intravenous immunoglobulin (IVIG) (2g/kg) and aspirin (80mg/kg/day) were given during hospital admission. Resolution of fever and conjunctival injection were achieved during 48 hours after initiation of IVIG. Liver enzymes normalized within 1 week from the start of the febrile illness and before IVIG. Platelets count rose to 900,000/mm^3^ after 2 weeks from the onset of the illness. His echocardiographic findings were normal during his follow-up visits and aspirin therapy was continued on 5mg/kg/day.

*Why this presentation is an incomplete form of KD?*

- High-grade fever and jaundice were the main presentations
- Clinical criteria were incomplete: the presence of only 2 clinical findings; desquamation on the tips of fingers and bilateral bulbar conjunctival injection.
- Laboratory criteria helped to establish the diagnosis: evidence of hypoalbuminemia, elevated alanine aminotransferase and thrombocytosis.

## Discussion

An incomplete presentation of KD has been reported in 15 to 36.2% of patients^10,11^. Relatively more children with incomplete presentation were in the extremes of the age spectrum (<1 year old, or >5 to 9 years old)^10,12^. The frequently reported findings which are less frequently observed in incomplete presentation are cervical lymphadenopathy (19-38.6%) and extremity changes (21-44.5%)^10–13^. Sudo et al. concluded that the higher incidence of coronary artery lesions in patients with incomplete presentation was firstly due to diagnostic bias because of the use of echocardiography in the diagnostic process, and secondly due to delays in the treatment because of difficulties in making the diagnosis^14^. In children with incomplete KD, the time between onset of symptoms and diagnosis has been reported to be longer^12,14,15^. Diagnosis of KD can be challenging in the absence of a confirmatory test or pathognomonic finding, especially when clinical criteria are incomplete. Unfortunately, the majority of published papers describing possible biomarkers for KD have used inappropriate control groups as the comparator. The appropriate controls for such studies should be febrile children with some clinical signs that are shared with KD such as rash or conjunctival injection. Until such time as appropriately designed studies are performed, we must maintain a high index of suspicion of KD in children presenting with unexplained fever.

## Conclusions

1- It is important for the treating physicians to become aware of cases of incomplete KD as prompt diagnosis and early treatment of these patients with intravenous immunoglobulin is vital for the prevention of potentially lethal coronary complications.
2- High index of suspicion is required for early diagnosis and management of Kawasaki disease irrespective of the clinical presentation. Early diagnosis can result in improved outcomes.

## References

[1] Cohen E, Sundel R (2016). Kawasaki disease at 50 years. JAMA Pediatr.

[2] Kawasaki T (1967). Acute febrile mucocutaneous syndrome with lymphoid involvement with specific desquamation of the fingers and toes in children. Arerugi.

[3] Zhu FH, Ang JY (2016). The clinical diagnosis and management of Kawasaki disease: A review and update. Curr Infect Dis Rep.

[4] Tizard EJ (2005). Complications of Kawasaki disease. Current Pediatrics.

[5] McCrindle BW, Rowley AH, Newburger JW, Burns JC, Bolger AF, Gewitz M, Baker AL, Jackson MA, Takahashi M, Shah PB, Kobayashi T, Wu MH, Saji TT, Pahl E, E. American Heart Association Rheumatic Fever, Y. Kawasaki Disease Committee of the Council on Cardiovascular Disease in the, C. Council on, N. Stroke, S. Council on Cardiovascular, Anesthesia, E. Council on and Prevention (2017). Diagnosis, treatment, and long-term management of Kawasaki disease: A scientific statement for health professionals from the American heart association. Circulation.

[6] Verma P, Agarwal N, Maheshwari M (2015). Recurrent Kawasaki disease. Indian Pediatr.

[7] Sonobe T, Kawasaki T (1987). Atypical Kawasaki disease. Prog Clin Biol Res.

[8] Barone SR, Pontrelli LR, Krilov LR (2000). The differentiation of classic Kawasaki disease, atypical Kawasaki disease and acute adenoviral infection: Use of clinic features and a rapid direct fluorescent antigen test. Arch Pediatr Adolesc Med.

[9] Forsey J, Mertens L (2011). Atypical Kawasaki disease - A clinical challenge. Eur J Pediatr.

[10] Sonobe T, Kiyosawa N, Tsuchiya K, Aso S, Imada Y, Imai Y (2007). Prevalence of coronary artery abnormality in incomplete Kwasaki disease. Pediatr Int.

[11] Perrin L, Letierce A, Guitton C, Tran TA, Lambert V, Kone-Paut I (2009). Comparative study of complete versus incomplete Kawasaki disease in 59 pediatrics patients. Joint Bone Spine.

[12] Manlhiot C, Christie E, Mc Crindle BW, Rosenberg H, Chahal N, Yeung RS (2011). Complete and incomplete Kawasaki disease: Two sides of the same coin. Eur J Pediatr.

[13] Yellen ES, Gauvreau K, Takahashi M, Burns JC, Shulman S, Baker AL (2010). Performance of 2004 American Heart Association recommendations of treatment of Kawasaki disease. Pedaitrics.

[14] Sudo D, Monobe Y, Yashiro M, Mieno MN, Uehara R, Tsuchiya K (2012). Coronary artery lesions of incomplete Kawasaki disease: A nationwide survery in Japan. Eur J Pediatr.

[15] Witt MT, Minich LL, Bohnsack JF, Young PC (1999). Kawasaki patients are being diagnosed who do not meet American Heart Association criteria. Pediatrics.

